# Genome Sequence Resource of the Avocado Scab Pathogen Elsinoe perseae

**DOI:** 10.1128/mra.00190-23

**Published:** 2023-05-16

**Authors:** Lederson Gañán-Betancur, Romina Gazis

**Affiliations:** a Department of Plant Pathology, University of Florida, Homestead, Florida, USA; b Tropical Research and Education Center, University of Florida, Homestead, Florida, USA; Vanderbilt University

## Abstract

We present a draft genome sequence of Elsinoe perseae, an economically important plant pathogen of commercially grown avocados. The 23.5-Mb assembled genome consists of 169 contigs. This report represents an important genomic resource to guide future research aimed at understanding the genetic interactions of *E. perseae* with its host.

## ANNOUNCEMENT

The fungus Elsinoe perseae (Dothideomycetes: Myriangiales) causes avocado scab, a major disease affecting avocado (Persea americana) production across several countries. Like other *Elsinoe* species, *E. perseae* can synthesize elsinochrome (1), a type of photoactivated perylenequinone toxin suggested to play an important role in host pathogenesis (2). To support future research on avocado scab, we sequenced a genome of *E. perseae*, presented here.

Leaves with typical scab lesions, as described by Jenkins (3), were collected from avocado trees (cv. Lima Late) growing in the tropical fruit germplasm collection at the University of Florida’s Tropical Research and Education Center. Small leaf fragments (5 by 5 mm) were cut and surface sterilized by immersion in 0.8% NaCl solution (1 min), 70% ethanol solution (1 min), and sterile distilled water (3 min). The leaf fragments were laid onto the surface of potato dextrose agar medium (PDA; Difco Laboratories) and incubated for 7 days at 25°C in darkness. A fungal colony culture arising from one of the scab lesions was used to obtain a monoconidial strain, designated TREC-ASLL3. The fungal strain was maintained on PDA at 25°C in darkness. Genomic DNA was isolated from 2-week-old fungal colonies using the E.Z.N.A. high-performance (HP) fungal DNA kit (Omega Bio-Tek, Georgia) following the manufacturer’s instructions. To confirm the species identity, the internal transcribed spacer (ITS) locus was sequenced using the primer pair ITS5/ITS4 (4) and used in a BLASTn search of GenBank with default parameters. Based on 99% nucleotide similarity (613/615 bp) to the reference type strain *E. perseae* CBS 406.34 (GenBank accession number KX887258), the species identity was confirmed. Genomic DNA of *E. perseae* TREC-ASLL3 was then processed using the NEBNext Ultra DNA library prep kit for Illumina (New England Biolabs, Ipswich, MA), and the resulting library was sequenced (2 × 150 bp) using the NovaSeq 6000 platform (Illumina, San Diego, CA), producing a total of 7,223,594 read pairs. The Illumina read quality was checked using FastQC v0.11.9 (5), and adaptors and low-quality reads (<Q20) were removed using Trimmomatic v0.38 (6). The reads that passed the quality control procedures were assembled using MEGAHIT v1.2.9 (7), and the quality of the resulting assembly was assessed using QUAST v5.2.0 (8). BUSCO v5.4.4 software (9) was used to assess the genome completeness with the ascomycota_odb10 data set. Default parameters were used during the assembly process. Summary statistics of the assembly are provided in Table 1.

**TABLE 1:** Summary statistics for the genome assembly of *Elsinoe perseae* strain TREC-ASLL3

Genome assembly feature	Value
GenBank accession no.	
Genome	JARDAA000000000
Version	JARDAA010000000
Assembly statistics	
Total length (bp)	23,492,853
No. of contigs (≥1,000 bp)	169
Size of largest contig (bp)	2,124,429
*N*_50_ (bp)	561,242
*L*_50_ (contig)	14
GC content (%)	52.35
Completeness (% of complete and single-copy BUSCOs)^*a*^	98.00

a BUSCO analysis was based on a set of 1,706 fungal orthologs (data set: ascomycota_odb10).

Genes involved in secondary metabolite production were identified using antiSMASH v7 (10) by comparison against the MIBiG (Minimum Information about a Biosynthetic Gene cluster) database of manually curated biosynthetic gene clusters (BGCs). One of the predicted BGCs contained a putative polyketide synthase gene, designated EPePKS1, that showed 57% sequence similarity to a polyketide synthase from the known BGC elsinochrome C (MIBiG accession number BGC0001865 from Parastagonospora nodorum).

Genomic resources for the genus *Elsinoe* are scarce and limited to species that cause economically significant damage to cultivated crops. The work presented here will contribute to the better understanding of the *E. perseae* and *P. americana* pathosystem and provide resources for future comparative analysis at the genus level.

### Data availability.

This whole-genome shotgun project has been deposited at DDBJ/ENA/GenBank under the accession number JARDAA000000000 (BioProject accession number PRJNA938760). The version described here is JARDAA010000000. The corresponding raw reads from the Illumina sequencing are available in the SRA under the accession number SRR23619844. The partial sequence of the ITS region was deposited at GenBank under accession number OQ526214. Additional data sets generated or analyzed during the current study, including the complete sequence of the putative polyketide synthase EPePKS1, are publicly available at the Open Science Framework repository (https://osf.io/bdmye/). A culture of strain *E. perseae* TREC-ASLL3 was deposited at the ARS (NRRL) Culture Collection (Peoria, IL) under the accession number NRRL 64475.
